# Effect of Germination and Subsequent Fermentation Period on the Functional, Color, and Antinutrients of Pigeon Pea ( *Cajanus cajan* ) Flour and Its Structural Parts

**DOI:** 10.1002/fsn3.70750

**Published:** 2025-07-31

**Authors:** Sunday Samuel Sobowale, Bukola Funmilola Olatunji, Olanrewaju Emmanuel Fayemi

**Affiliations:** ^1^ Department of Food Science and Technology, College of Basic and Applied Sciences Mountain Top University Ibafo Ogun State Nigeria; ^2^ Department of Biological Sciences, College of Basic and Applied Sciences Mountain Top University Ibafo Ogun State Nigeria

**Keywords:** *Cajanus cajan*, fermentation, functional properties, germination

## Abstract

This study assessed the impact of germination and fermentation on the nutritional composition, physicochemical, functional, and sensory quality of pigeon pea flour from various grain fractions. Treatments applied between 24 and 72 h led to notable improvements in protein content, with germinated‐fermented bran flour at 72 h reaching 21.20% compared to 12.62% untreated whole grain flour. Fat and ash contents also increased with treated samples, whereas carbohydrate content decreased with extended processing. Functional properties showed significant shifts, with water absorption capacity increasing in the germinated‐fermented whole flour for 72 h (3.73 g/g), suggesting improved hydration behavior, whereas oil absorption increased to 1.43 g/g in the same sample, useful for flavor retention in formulated foods. Swelling power and solubility index also increased, particularly in the germinated‐fermented bran flour for 72 h, supporting potential use in soups and instant meals. Foaming capacity improved in several treated flours, peaking at 23.5%, which may benefit baked or whipped products. Dispersibility values above 60% in certain samples indicate reconstitution potential for powdered applications. Acidification during fermentation lowered pH and increased titratable acidity, supporting shelf stability. Color parameters shifted toward darker, less red hues, influenced by treatment type and duration. Antinutrient levels, including tannins and oxalates, reduced significantly, enhancing nutritional quality. Sensory scores favored germinated‐fermented dehulled grain at 48 h and germinated‐fermented rootless flour for 72 h. Overall, germination and fermentation improved the functionality and nutrient density of pigeon pea flour, supporting its integration into high‐protein, ready‐to‐use food products.

## Graphical abstract

This study investigates the impact of germination and fermentation periods (24, 48, and 72 h) on the quality attributes of pigeon pea flour and its structural parts. Germination and fermentation of pigeon pea possess good properties that are suitable for inclusion in gluten‐free, as well as in the development of ready‐to‐use food formulations. This study highlights the potential of germinated and fermented pigeon pea flour in addressing protein‐energy malnutrition and in developing infant and baked food products.

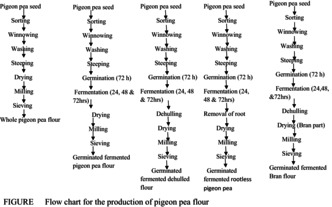

## 1 Introduction

Pigeon pea ( *Cajanus cajan* ) is one of the edible legumes in the world, and it is a crop that grows well in Africa (Fatokimi and Tanimonure 2021). Pigeon pea is regarded as an underutilized grain legume that grows in the tropics and subtropics (Sarkar et al. 2020). Their varieties consist of protein content ranging from 18% to 30%, making them an important source of dietary protein for over a billion people worldwide (Obala et al. 2019; Singh et al. 2020). Although the protein content is comparable to that of other legumes, such as cowpea and groundnut, it has low protein digestibility (Sun et al. 2020). Aside from protein, pigeon pea is also a rich source of fiber, minerals, vitamins (including riboflavin, thiamine, choline, and niacin), and antioxidants (Babarinde et al. 2020). In addition to its nutritional value, pigeon pea also possesses various medicinal properties due to its high content of polyphenols and flavonoids (Singh, Gupta, and Singh 2016). It is an integral part of traditional folk medicine in India, China, and some other nations (Saxena et al. 2010). Fresh seeds of pigeon pea are used in managing urinary incontinence in men, whereas immature seeds have been recommended for the treatment of kidney ailments. Its seeds are often combined with coffee to alleviate headaches and vertigo (Saxena et al. 2010). Furthermore, dried roots are utilized for their alexipharmic, anthelmintic, expectorant, sedative, and therapeutic properties (Saxena et al. 2010). Pigeon pea contains antinutrients such as tannins, cyanogenic glycosides, hemagglutinin, and alkaloids that can reduce the bioavailability of nutrients such as proteins (Aruna and Devindra 2016). It also contains flatulent factors, which are present in oligosaccharides such as stachyose, verbascose, and raffinose (Elango et al. 2022). However, due to its tough texture, hard‐to‐cook nature, and the presence of antinutrients, its utilization is limited (Fasoyiro and Arowora 2013). Several researchers have employed various approaches to improve the food value chain of pigeon peas, particularly in their processing, storage, preservation, and utilization. These include germination, fermentation, toasting (Onimawo and Akubor 2012), and boiling and irradiation processes (Onimawo and Akubor 2012). The choice of processing methods employed in the preparation can significantly influence the physicochemical properties of the seeds, thereby affecting their potential applications in food products.

Fermentation is a bioprocess that utilizes microorganisms and their enzymes to develop desirable quality characteristics in food products (Singhania et al. 2009; Odion‐Owase et al. 2018). According to Nkhata et al. (2018), fermentation improves food digestibility and nutritional quality by increasing the bioavailability of nutrients in crops while also improving taste. It alters the intestinal microflora balance and inhibits the growth of harmful bacteria, promotes good digestion, boosts immune function, and increases resistance to infection (Helland et al. 2004; Odion‐Owase et al. 2018). Germination (malting), on the other hand, involves carefully sprouting seeds to induce a specific desired change in their physical and biochemical composition, which is subsequently stabilized through drying (Gupta et al. 2010). The primary aim of malting is to stimulate the development of hydrolytic enzymes, which are absent in ungerminated grains (Baranwal 2017). The process consists of three steps: steeping, germination, and drying (kilning).

Due to the increasing demand for affordable and readily available plant protein sources to meet the nutritional needs of the African population, many studies have been conducted to enhance the utilization of pigeon pea in human diets (Sakyi‐Dawson et al. 2016; Sobowale et al. 2025). Studies have also shown that improving the dehulling process and adding potash during cooking can reduce cooking time and increase the acceptability of pigeon peas (Momoh et al. 2019; Fasoyiro et al. 2010). Furthermore, fermentation has been reported to improve the protein content and other qualities of the seeds (Adebowale and Maliki 2011), whereas malting has been found to significantly enhance the nutritional quality and digestibility of pigeon pea and reduce its antinutritional composition (Asouzu and Umerah 2020; Sharma et al. 2019; Nwosu et al. 2013). However, despite the individual records of these processes, the potential synergistic effect of combining germination and fermentation has not been thoroughly investigated. Therefore, this study aims to evaluate the impact of fermentation and germination on the various structural, proximate, functional, and antinutritional properties of pigeon pea flour.

## 2 Materials and Methods

### 2.1 Raw Materials and Sample Preparation

The mature white pigeon pea ( *Cajanus cajan* ) used in this study was purchased from the local market in Lagos, Nigeria. The seeds were sorted to remove dirt, stones, and other impurities, then winnowed. They were thoroughly washed with clean water, after which the seeds were divided into five equal portions of 2 kg each. The first portion was processed fresh (untreated), while the other four portions were subjected to different processing treatments.

#### 2.1.1 Processing of Whole Pigeon Pea Flour

**FIGURE 1:**
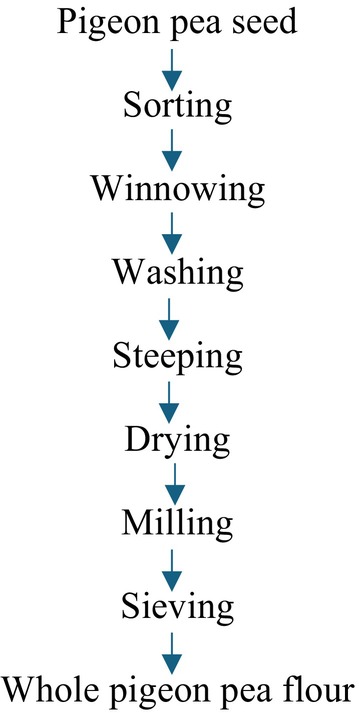
Processing of whole pigeon pea flour.

The method of Nwosu et al. (2013) was employed for processing pigeon pea flour, as presented in Figure 1. Raw pigeon pea seeds were cleaned by sorting out dirt and stones and then washed to remove any remaining dirt. The seeds were oven‐dried (Tempo Laboratory Oven) at 60°C for 12 h and then milled into fine flour using an attrition mill. After sieving through an 80‐μm mesh sieve, they were stored in air‐tight, high‐density polyethylene bags for further analysis.

#### 2.1.2 Processing of Germinated‐Fermented Pigeon Pea Flour

**FIGURE 2:**
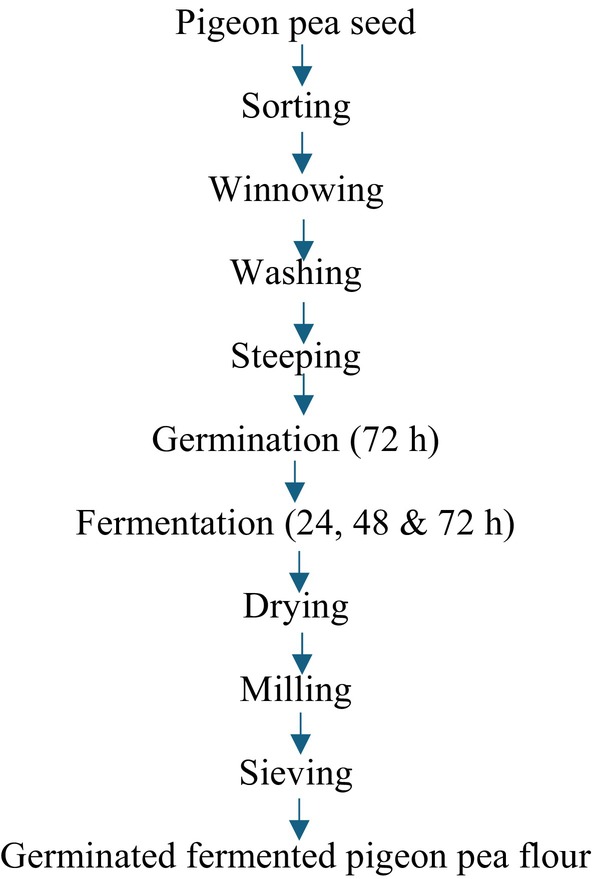
Processing of germinated‐fermented pigeon pea flour.

**FIGURE 3:**
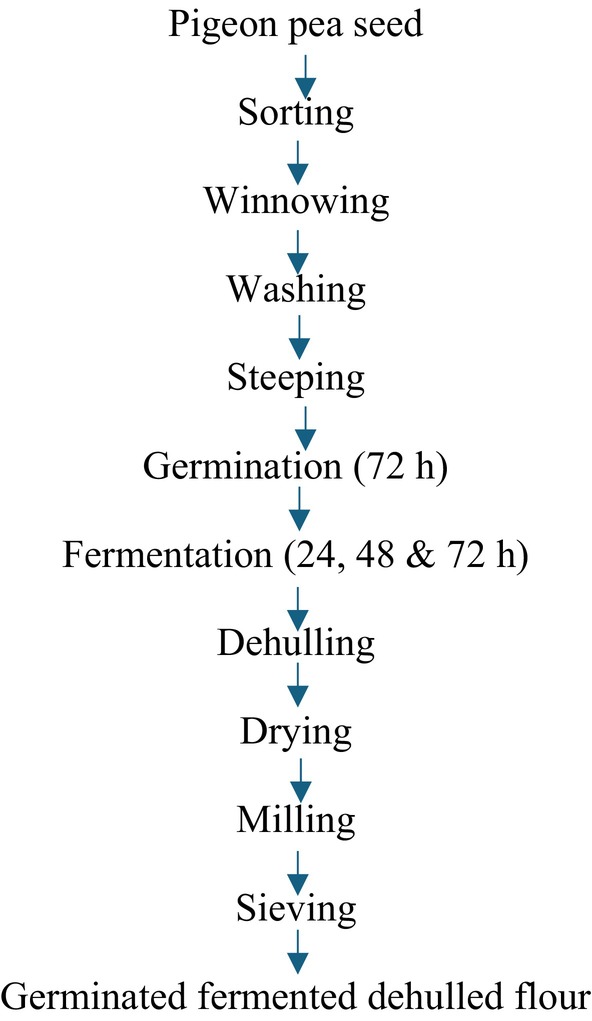
Processing of germinated‐fermented dehulled pigeon pea flour.

**FIGURE 4:**
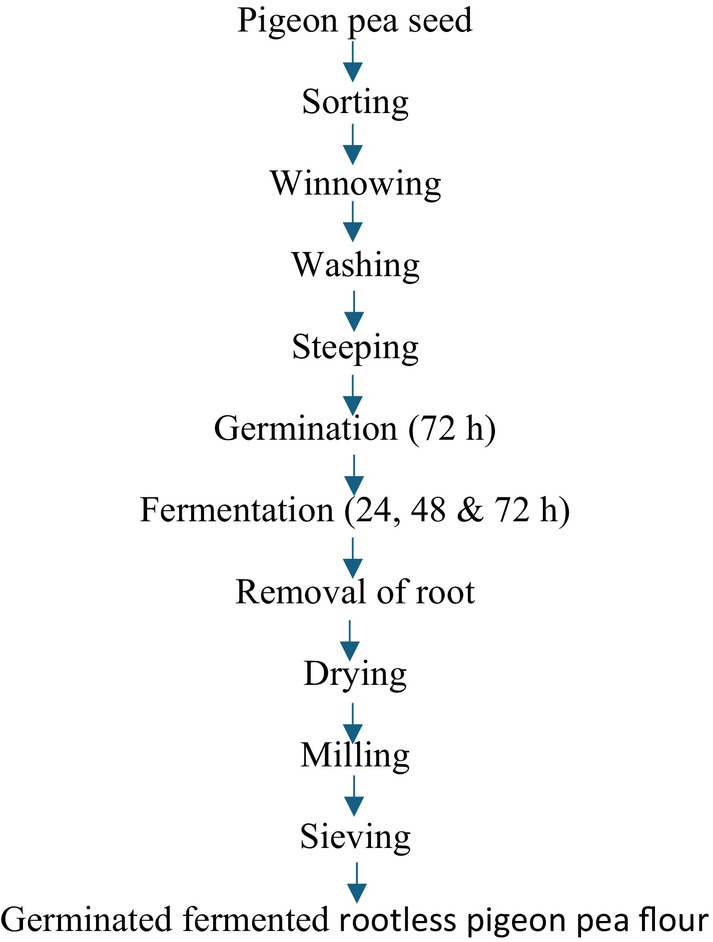
Processing of germinated‐fermented rootless pigeon pea flour.

**FIGURE 5:**
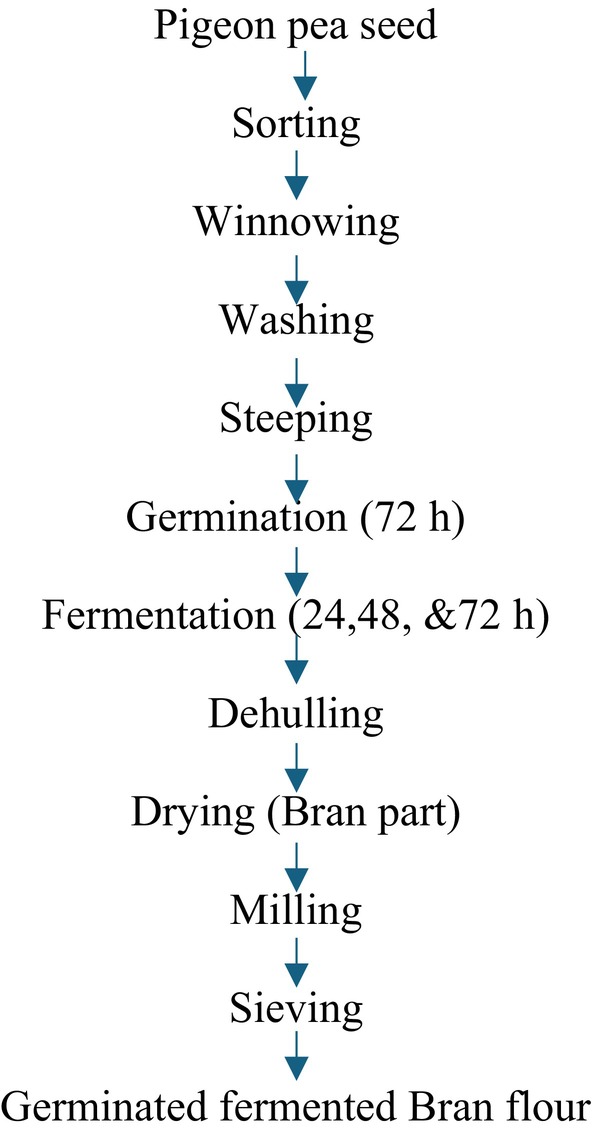
Processing of germinated‐fermented bran pigeon pea flour.

The method of Nwosu et al. (2013) with slight modifications was used in the processing of pigeon pea flour. Two kilograms of each sorted white pigeon pea sample were rinsed thoroughly with tap water and soaked for 12 h in water. The hydrated seeds were drained and spread on layers of wet jute bags and were allowed to germinate for 3 days in the dark. The jute bags were moistened at regular 12‐h intervals to facilitate germination. At the end of germination, the ungerminated seeds from each pigeon pea sample were discarded, and the germinated seeds were rinsed and subsequently fermented using solid state fermentation for 0, 24, 48, and 72 h. Each germinated pigeon pea sample was put in a cleaned container and soaked with cleaned water, properly sealed, and allowed to undergo fermentation at ambient temperature (30°C) for 3 days. The fermentation process was terminated for each sample; the germinated‐fermented whole flour (GFWG) was processed as shown in Figure 2, germinated‐fermented dehulled pigeon pea flour (GFPG) was processed as shown in Figures 3 and 4, and the process of germinated‐fermented rootless pigeon pea (GFRF) and germinated‐fermented bran flour (GFBF) was processed as shown in Figure 5. All the samples were oven‐dried (Tempo Laboratory Oven) at 60°C for 12 h, milled into a fine powder using an attrition mill (Model 200 L090; E.H. Bentall, UK), sieved through an 80‐μm mesh sieve, and stored in air‐tight, high‐density polyethylene bags for further analysis.

### 2.2 Proximate Composition

The moisture, protein, fat, crude fiber, and ash contents of the samples were determined according to the procedure described by AOAC International (2012). Protein content was determined using the Kjeldahl method and calculated by multiplying the nitrogen percentage by a factor of 6.25. Carbohydrate was determined by differences (%).

### 2.3 Physicochemical Properties

The use of a pH meter determined the pH of samples (JENWAY model 3520; Bibby Sci. Ltd., Essex, UK) while the total titratable acidity (TTA) was determined using the methods as described by AOAC (2010).

### 2.4 Functional Properties

#### 2.4.1 Bulk Density

The method described by Ikechukwu et al. (2017) was used to determine the bulk density. Briefly, the flour sample (20 g) was weighed into a 50 mL graduated measuring cylinder. The cylinder was tapped gently against the palm until a constant volume was obtained. Bulk density was calculated as:

$$\text{Bulk density}\;\left(g/\mathrm{mL}\right)=\frac{\text{Weight of sample}}{\text{Volume of sample after tapping}}$$

#### 2.4.2 Water Absorption Capacity

The water absorption capacity (WAC) of the samples was determined using the method described by Omowaye‐Taiwo et al. (2015). Ten milliliter of distilled water was added to 1 g of each flour sample; the suspension was stirred using a magnetic stirrer for 3 min. The suspension was transferred into centrifuge tubes and centrifuged at 3500 rpm for 30 min. The supernatant obtained was measured using a 10 mL measuring cylinder. The density of the water was assumed to be 1 g/mL. The water absorbed by the sample was calculated as the difference between the initial water used and the volume of the supernatant obtained after centrifuging. The result was expressed as a percentage of water absorbed by the blends on a weight‐to‐weight (g/g) basis.

$$\%\mathrm{WAC}=\frac{\text{Weight of water absorbed}\times \text{density of water}\;}{\text{weight of sample}}\times 100$$

#### 2.4.3 Oil Absorption Capacity

The oil absorption capacity (OAC) of the samples was determined using the method described by Omowaye‐Taiwo et al. (2015). About 10 ml of oil of known specific gravity was added to 1 g of the sample in a beaker. The suspension was stirred using a magnetic stirrer for 3 min. The suspension was then centrifuged at 3500 rpm for 30 min, and the supernatant was transferred into a 10 mL graduated cylinder. The density of oil was taken to be 0.931 g/mL. The oil absorbed by the flour was calculated as the difference between the initial volume of the oil and the volume of the supernatant.

$$\%\mathrm{OAC}=\frac{\text{Weight of oil absorbed}\times \text{density of oil}\;}{\text{weight of sample}}\times 100$$

#### 2.4.4 Swelling Index

The swelling index of the samples was determined using the method described by Awolu et al. (2017). Ten grams of the flour sample were weighed and poured into a 100 mL measuring cylinder, and the initial volume was taken. Sixty milliliters of water were then added and allowed to stand for 4 h after stirring. The level of swelling was then measured. $A=\pi r^{2}$ was recorded.

$$\text{Swelling index}=\frac{\text{volume after soaking}-\text{volume before soaking}}{\text{weight of sample}}\times 100$$

#### 2.4.5 Foaming Capacity and Stability

Foaming capacity (F.C.) and stability (F.S.) were determined according to the method described by Sobowale, Bamidele, and Adebo (2024). The flour sample was mixed thoroughly in a measured quantity of distilled water using a magnetic stirrer for approximately 5 min and then transferred to a graduated measuring cylinder. The volume of the foam measured was used to calculate the foaming capacity, as shown below:

$$F.C.\left(\%\right)=\frac{\text{Volume after homogenization}-\text{volume before homogenization}}{\text{volume before homogenization}}\times 100$$

The foam volume that remained after a 10‐min interval for 1 h was used to calculate the foaming stability, as shown below:

$$F.S.\left(\%\right)=\text{Volume of foam remained}-\text{initial volume of foam}\times 100$$

### 2.5 Color Characteristics

The color characteristics of pigeon pea flours (L*, a*, b*, and ∆*E*) were measured using a colorimeter (Konica Minola Camera Co., Osaka, Japan) and the procedure outlined by the International Commission on Illumination. The colorimeter was calibrated before sample analysis, and the samples were tested for lightness (L*), redness/greenness (a*), yellowness/blueness (b*), and total color differences (∆E*). All measurements were performed in triplicate, and the mean value was recorded.

### 2.6 Antinutrient Composition

#### 2.6.1 Tannin

The tannin content of the sample was determined using the Folin‐Denis Colorimetric method, as reported by Nwosu et al. (2014). One gram of the processed sample was mixed with distilled water in a 1:10 (w/v) ratio. The mixture was agitated for 30 min at room temperature and filtered to obtain the extract. A standard tannic acid solution was prepared. Two milliliters of the standard solution and an equal volume of distilled water were dispensed into separate 50 mL volumetric flasks, serving as the standard and reagent blank, respectively. About 2 mL of each sample extract was placed in its respective labeled flask. The content of each flask was mixed with 35 mL of distilled water, and 1 mL of the Folin‐Denis reagent was added to each. This was followed by 2.5 mL of saturated Na_2_CO_3_ solution. Thereafter, each flask was diluted to the 50 mL mark with distilled water and incubated for 90 min at room temperature. Their absorbance was measured at 710 nm using a colorimeter (Jenway 6051), with the reagent blank set to zero. The tannin content was calculated as shown below:

$$\%\text{Tannin}=100/W\times \frac{a_{u}}{a_{s}}\times C\times \frac{V_{t}}{V_{a}}\times D$$

where *W* = weight of sample, *a*_u_ = absorbance of test sample, *a*_s_ = absorbance of standard tannin solution, *C* = concentration of standard tannin solution, *V*_t_ = total volume of extract, *V*_a_ = volume of extract analyzed, *D* = dilution factor.

#### 2.6.2 Phytate

The method described by Nwosu (2011) was used to determine phytate. Briefly, the phytic acid in the samples was precipitated with excess FeCl_3_ after extracting 1 g of each sample with 100 mL of 0.5 N HCl. The precipitate was converted to sodium phytate using 2 mL of 2% NaOH before digestion with an acid mixture containing equal portions (1 mL) of concentrated hydrochloric acid, H_2_SO_4_, and 65% HCl. The solution was filtered with filter paper, and 5 mL of 5% molybdate solution was added. It was allowed to stand for 30 min to allow for color development through the formation of a phosphomolybdate complex. The absorbance of the solution was measured colorimetrically (Jenway 6051 Colourimeter) at 520 nm after color development with a molybdate solution. The percentage phytate was thus calculated:

$$\%\text{Phytate}=100\;W_{t}\times a_{u}\times a_{s}\times C\times V_{t}\;V_{a}$$

where *W*_t_ = weight of sample used, *a*_u_ = absorbance of test sample, *a*_s_ = absorbance of standard phytate solution, *C* = concentration of standard phytate solution, *V*_t_ = total volume of extract, *V*_a_ = volume of extract analyzed.

#### 2.6.3 Oxalate

This was carried out by the procedures described by Nwosu et al. (2014). About 1 g of the sample was weighed into a 100‐mL beaker and 20 mL of 0.30 N HCl was added and warmed to 40°C–50°C using a magnetic hot plate and stirred for 1 h. It was extracted three times using a 20‐mL flask. The combined extract was diluted to the 100 mL mark of the volumetric flask. The oxalate was estimated by pipetting 5 mL of the extract into a conical flask and making it alkaline with 1.0 mL of 5 N ammonium hydroxide. A little indicator paper was placed in the conical flask to enable one to know the alkaline regions. It will also be made acidic to phenolphthalein (three drops of this indicator added; excess acid decolorizes the solution) by dropwise addition of glacial acetic acid. Approximately 1 mL of 5% CaCl_2_ was then added, and the mixture was allowed to stand for 3 h. It was subsequently centrifuged at 300 rpm for 15 min. The supernatant was discarded, and about 2 mL of 3 N H_2_SO_4_ was added to each tube, and the precipitate was dissolved by warming in a water bath (70°C–80°C). The contents of all the tubes were carefully poured into a clean conical flask and titrated with freshly prepared 0.01 N KMnO_4_ at room temperature (28°C ± 2°C) until a pink color appeared throughout the solution. It was allowed to stand until the solution became colorless. The solution will then be warmed to 70°C–80°C and titrated until a permanent pink color that persists for at least 30 s is attained. The percentage (%) oxalate content will thus be calculated:

%Oxalate=100W×0.00225×total titer volume

where *W* = weight of sample used.

### 2.7 Statistical Analysis

All analyses were conducted in triplicate, and the data obtained were subjected to one‐way analysis of variance (ANOVA) using Statistical Package for Social Sciences (SPSS, Version 20) software. Significant means were separated using Duncan's New Multiple Range Test (DNMRT). Differences were considered significant at *p* < 0.05, and results were expressed as mean ± standard deviation.

## 3 Results and Discussion

### 3.1 Proximate Composition of Germinated and Fermented Pigeon Pea Flour

The proximate composition of fermented and germinated pigeon pea flour samples is presented in Table 1. Proximate composition is an accurate representation of the nutritive value of any food, used to define its nutritional content (Aja et al. 2015). The moisture content of the flour samples ranged from 6.10% to 12.08%. The samples with germinated‐fermented whole grain for 72 h (GFWG72) and germinated‐fermented whole grain for 24 h (GFWG24) had the highest and lowest values, respectively. The moisture content of the unfermented samples did not show significant differences (*p* > 0.05). Moisture content in a food sample serves as an indicator of stability, influencing appearance, shelf life, and product yield (Obinna‐Echem et al. 2019). Germination led to an increase in moisture content, as unfermented‐germinated whole grain (UGWG) samples (8.58%) exhibited higher moisture levels than whole grain flour (WGF) samples (8.32%). This suggests that germination causes an increase in moisture due to the seed's absorption of water. The low moisture content of the samples is desirable, falling within the acceptable limits for flours (< 14%). The low moisture value is in close agreement with the value of 6.39% reported for pawpaw seeds (Fayemi et al. 2023) and sprouted and non‐sprouted watermelon seeds as reported by Olatidoye et al. (2021), but lower than the value reported for cowpea (10.39%) by Masood and Rizwana (2010). Additionally, low moisture indicates a higher dry solids content, inactivates enzymatic activities, and controls microbial growth (Obinna‐Echem et al. 2019).

**TABLE 1:** Proximate composition of germinated‐fermented pigeon pea flour samples.

Samples	Moisture (%)	Protein (%)	Fat (%)	Ash (%)	Crude fiber (%)	Carbohydrate (%)
UWGF	8.32^efgh^ ± 0.16	12.62^l^ ± 0.25	1.34^h^ ± 0.04	3.25^cdef^ ± 0.36	7.72^ab^ ± 0.87	68.50^bc^ ± 1.03
WGFF24	6.10^k^ ± 0.22	10.87^n^ ± 0.00	3.74^b^ ± 0.27	4.73^bc^ ± 0.35	6.87^b^ ± 1.25	67.33^cd^ ± 1.49
WGFF48	6.59^jk^ ± 0.19	15.95^i^ ± 0.25	1.90^fg^ ± 0.17	3.18^ef^ ± 1.07	8.33^a^ ± 0.39	65.52^de^ ± 0.55
WGFF72	7.45^hi^ ± 0.70	18.23^ef^ ± 0.00	1.67^fgh^ ± 0.17	1.71^g^ ± 0.33	7.75^ab^ ± 0.69	60.33^ij^ ± 0.95
UGWG	8.58^ef^ ± 0.37	19.80^c^ ± 0.25	1.60^fgh^ ± 0.06	8.06^a^ ± 0.39	2.31^cdef^ ± 0.15	69.62^ab^ ± 1.26
GFWG24	7.68 ^fghi^ ± 0.81	16.91^h^ ± 0.12	1.79^fg^ ± 0.05	4.41^cde^ ± 0.69	1.40^fgh^ ± 0.11	65.68^de^ ± 1.50
GFWG48	8.79^de^ ± 0.52	17.61^g^ ± 0.12	1.54^gh^ ± 0.10	2.25^fg^ ± 0.35	1.71^efg^ ± 0.22	62.39^h^ ± 0.06
GFWG72	12.08^a^ ± 0.35	18.14^f^ ± 0.12	4.06^b^ ± 0.41	1.94^fg^ ± 0.68	1.10^efg^ ± 0.12	59.66^jk^ ± 0.30
UGPG	8.94^de^ ± 0.24	19.28^d^ ± 0.25	2.05^f^ ± 0.05	4.40^cde^ ± 0.68	1.27^gh^ ± 0.08	68.28^bc^ ± 1.21
GFPG24	8.26^efgh^ ± 0.43	13.50^k^ ± 0.25	2.48^e^ ± 0.14	3.92^cde^ ± 0.67	2.19^cdefg^ ± 0.10	67.85^bc^ ± 0.52
GFPG48	8.12^efghi^ ± 0.09	15.34^j^ ± 0.12	3.79^b^ ± 0.24	3.88^cde^ ± 0.17	2.59^cde^ ± 0.05	64.56^ef^ ± 0.70
GFPG72	11.13^b^ ± 0.26	20.42^b^ ± 0.12	2.78^de^ ± 0.17	3.73^cde^ ± 1.06	3.10^c^ ± 0.21	58.18^k^ ± 0.43
UGRF	8.37^efgh^ ± 0.65	18.40^ef^ ± 0.25	3.24^c^ ± 0.35	4.70^bcd^ ± 0.35	2.34^cdef^ ± 0.49	62.95^fgh^ ± 0.89
GFRF24	10.16^c^ ± 0.56	16.65^h^ ± 0.25	2.52^e^ ± 0.11	4.73^bc^ ± 0.38	1.82^efg^ ± 0.14	64.71^ef^ ± 0.01
GFRF48	7.51^ghi^ ± 0.04	18.66^e^ ± 0.12	3.90^b^ ± 0.11	4.14^cde^ ± 0.33	2.23^cdefg^ ± 0.16	64.44^efg^ ± 0.25
GFRF72	7.26^ij^ ± 0.14	20.85^a^ ± 0.25	3.03^cd^ ± 0.19	3.73^cde^ ± 0.38	1.75^efg^ ± 0.14	61.90^hi^ ± 0.13
UBRF	8.94^de^ ± 0.24	19.28^d^ ± 0.12	3.32^c^ ± 0.09	2.94^efg^ ± 0.67	2.09^defg^ ± 0.01	62.64^gh^ ± 0.94
GFBRF24	7.67^fghi^ ± 0.36	11.84^m^ ± 0.12	3.31^c^ ± 0.28	5.97^b^ ± 1.36	0.71^h^ ± 0.02	70.50^a^ ± 1.17
GFBRF48	8.43^efg^ ± 0.14	20.15^bc^ ± 0.25	1.77^fgh^ ± 0.04	4.41^cde^ ± 0.03	2.06 ^efg^ ± 0.08	61.66 ^hi^ ± 0.18
GFBRF72	7.56^ghi^ ± 0.12	21.20^a^ ± 0.00	5.37^a^ ± 0.22	3.20 ± 0.35^def^	3.06^cd^ ± 0.23	61.13^hij^ ± 0.02

*Note:* Values with different superscript letters in the same column are significantly different (*p* < 0.05).

Abbreviations: GFBRF24, germinated‐fermented bran flour for 24 h; GFBRF48, germinated‐fermented bran flour for 48 h; GFBRF72, germinated‐fermented bran flour for 72 h; GFPG24, germinated‐fermented dehulled grain for 24 h; GFPG48, germinated‐fermented dehulled grain for 48 h; GFPG72, germinated‐fermented dehulled grain for 72 h; GFRF24, germinated‐fermented rootless flour for 24 h; GFRF48, germinated‐fermented rootless flour for 48 h; GFRF72, germinated‐fermented rootless flour for 72 h; GFWG24, germinated‐fermented grain for 24 h; GFWG48, germinated‐fermented grain for 48 h; GFWG72, germinated‐fermented grain for 72 h; UBRF, unfermented‐germinated bran flour for 0 h; UGPG, unfermented‐germinated dehulled grain for 0 h; UGRF, unfermented‐germinated rootless flour for 0 h; UGWG, unfermented‐germinated grain for 0 h; UWGF, unfermented‐germinated whole grain for 0 h; WGFF24, germinated‐fermented whole grain for 24 h; WGFF48, germinated‐fermented whole grain for 48 h; WGFF72, germinated‐fermented whole grain for 72 h.

Proteins play a crucial role in nutrition by catalyzing, regulating, protecting, and providing energy (Adamu et al. 2017). Crude protein obtained from germinated and fermented pigeon pea flour samples ranged between 10.87% and 21.20%. Significant differences (*p* < 0.05) exist among the samples, with germinated‐fermented bran flour for 72 h (GFBRF72) having the highest protein content (21.20%), while germinated‐fermented whole grain for 24 h (WGF24) has the lowest (10.87%) values. It was generally observed that the protein content of the samples increased with the duration of fermentation, which agrees with the findings of Osman (2011), Yousif and El Tinayi (2001), and Olatidoye et al. (2021) during their studies on the fermentation of pearl millet and sorghum, as well as the germination of watermelon seeds. The findings of this study also revealed that germinated‐fermented bran flour (GFBRF) exhibited a significant (*p* < 0.05) increase in protein concentration compared to other types, which can be further improved through fermentation. Germination also exhibited a positive relationship with protein, which is consistent with the findings of Laxmi et al. (2015). The increase in protein is attributed to the production of specific amino acids during protein synthesis (Chauhan et al. 2022).

Fat provides energy, essential fatty acids, and fat‐soluble vitamins (A, D, E, and K). It also enhances the palatability of food (Obinna‐Echem et al. 2019). The fat content of the samples varied significantly (*p* < 0.05), ranging from 1.34% to 5.37%. The sample GFBRF72 (5.37%) had the highest fat content, and the unfermented‐germinated whole grain flour (UWGF) (1.34%) had the lowest. The observed variation in the fat content of pigeon pea samples aligns with the submission of Nkhata et al. (2018), who stated that the fat content of cereals slightly increases during the steeping stage of germination but later declines during the germination phase, as well as lipids are used for the respiration process. Fats are essential to the human body because they provide the body with maximum energy and facilitate the absorption and transportation of fat‐soluble vitamins. The overall decrease in fat content aligns with previous studies, which reported a reduction in fat content during germination and fermentation (Sobowale, Otolowo, et al. 2024) and the sprouting of watermelon seeds (Olatidoye et al. 2021). This has been attributed to the hydrolysis and utilization of fats as an energy source for biochemical reactions during germination (Moongngarm and Saetung 2010; Jan et al. 2017), increased activities of lipolytic enzymes during fermentation, which hydrolyze fat components into fatty acids and glycerol (Chinma et al. 2009), and exhaustion of the fat stored during sprouting (Olatidoye et al. 2021).

The unfermented‐germinated whole grain sample (UGWG) indicates the highest ash value (8.06%), while the lowest value (1.71%) was observed in germinated‐fermented whole grain for 72 h (WGFF72). The results observed that there was no significant difference (*p* > 0.05) between the germinated‐fermented dehulled grain (GFPG) and germinated‐fermented rootless flour (GFRF) samples. Additionally, all samples fermented for 24 and 72 h showed significant differences. The ash content of the samples, including germinated‐fermented whole grain (GFWG), germinated‐fermented rootless flour (GFRF), and germinated‐fermented bran flour (GFBRF), increased rapidly at a fermentation duration of 24 h, after which the value decreased with an increase in the fermentation period. However, the ash content of the germinated‐fermented whole grain (WGFF) and germinated‐fermented grain (GFWG) decreased with a progressive increase in fermentation duration. The decrease in ash content was also observed by Obinna‐Echem et al. (2019) for germinated maize flour. Ash content provides an estimate of the mineral element concentration in the samples (Otori and Mann 2014).

The crude fiber content of the samples ranged from 0.71% to 8.33%, with germinated‐fermented whole grain for 48 h (WGFF48) exhibiting the highest fiber content (8.33%), and germinated‐fermented bran flour for 24 h (GFBRF24) having the lowest value (0.71%). A significant difference (*p* < 0.05) was observed among the samples. As the fermentation period increased, there was an initial decrease followed by an increase in crude fiber content (Table 1). Germination drastically reduced the crude fiber found in WGFF and GFWG samples. This finding contrasts with those of Rumiyati et al. (2012) and Jan et al. (2017), who reported that germination can be an effective method for improving the fiber content in foods. The change in crude fiber content could be attributed to the loss of dry matter resulting from the enzyme hydrolysis of starch and the microbial breakdown of cellular materials such as proteins, fats, and carbohydrates (Nkhata et al. 2018). The increase in fiber is desirable because fiber slows down the release of glucose from food, which could be beneficial for people with diabetes. Moreover, fiber forms gels in the stomach that slow down starch digestion and gastric emptying, subsequently increasing satiety (Yu et al. 2014; Nkhata et al. 2018). Carbohydrates (CHO) contain glucose, which the body needs for energy. They are the most important source of energy for the body (Aja et al. 2015). The carbohydrate content of germinated and fermented pigeon peas in the four samples varied between 58.18% and 70.50%. Significant differences (*p* < 0.05) exist among the samples, with GFBRF24 having the highest value (70.50%), while the lowest value (58.18%) was observed in germinated‐fermented peeled grain for 72 h (GFPG72). The carbohydrate content of the germinated samples decreased, while the carbohydrate content of the fermented samples increased with different durations. The decrease could be attributed to the enzymatic breakdown of carbohydrates into simple sugars through the activation of endogenous enzymes, such as α‐amylase, thereby improving the digestibility of complex carbohydrates. This occurs due to the degradation of starch, providing energy for seed development during malting and fermentation (Zhang et al. 2015; Oghbaei and Prakash 2016). Obinna‐Echem et al. (2019) reported a similar decrease in the carbohydrate content of germinated maize flour. Germination conditions have been reported to initiate the enzymatic activity of amylase and pullulanase, thereby hydrolyzing starch into smaller sugar molecules, which play a crucial role in nutrition by catalyzing, regulating, protecting, and providing energy (Adamu et al. 2017).

### 3.2 pH and Titratable Acidity of Germinated and Fermented Pigeon Pea Flour

The pH and TTA of germinated and fermented pigeon pea flour are shown in Table 2. The pH of the whole grain flour (WGF), germinated‐fermented grain (GFWG), germinated‐fermented peeled grain (GFPG), germinated‐fermented rootless flour (GFRF), and germinated‐fermented bran flour (GFBRF) ranged from 5.50 to 5.70, 5.30 to 5.70, 5.35 to 5.80, 5.35 to 5.65, and 5.45 to 5.85, respectively. The samples were significantly different (*p* < 0.05), with pH values ranging from 5.30 to 5.85. The pH provides a measure of the acidity strength in food. Therefore, the pH range indicates that the flours were acidic. The results also revealed that the pH of the samples decreased as the fermentation time increased. Samples fermented for 24 h were significantly different, except GFPG24 and GFRF24, which differed significantly from each other but showed no significant difference with the rest of the 24 h samples. A similar trend was observed in the 48 h samples, where no significant difference was found between the samples, except for WGFF48. Thus, the increase in acidity during fermentation may be due to organic acids produced during fermentation and related to the rate at which complex compounds are hydrolyzed (Gernah et al. 2011; Owheruo et al. 2019).

**TABLE 2:** pH and titratable acidity of germinated‐fermented pigeon pea flour samples.

Samples	pH	TTA (%)
UWGF	5.50^c^ ± 0.00	0.57^a^ ± 0.04
WGFF24	5.70^ef^ ± 0.00	2.07^j^ ± 0.04
WGFF48	5.30^a^ ± 0.00	1.35^fg^ ± 0.04
WGFF72	5.65^de^ ± 0.07	1.19^ef^ ± 0.21
UGWG	5.70^ef^ ± 0.00	0.68^ab^ ± 0.06
GFWG24	5.70^ef^ ± 0.00	0.81^bc^ ± 0.13
GFWG48	5.55^cd^ ± 0.07	1.65^hi^ ± 0.21
GFWG72	5.30^a^ ± 0.00	1.68^hi^ ± 0.04
UGPG	5.70^ef^ ± 0.00	0.81^bc^ ± 0.13
GFPG24	5.80^fg^ ± 0.14	1.08^de^ ± 0.00
GFPG48	5.55^cd^ ± 0.07	1.20^ef^ ± 0.00
GFPG72	5.35^ab^ ± 0.07	1.53^gh^ ± 0.00
UGRF	5.35^ab^ ± 0.07	1.08^de^ ± 0.00
GFRF24	5.65^de^ ± 0.07	1.46^gh^ ± 0.06
GFRF48	5.55^cd^ ± 0.07	1.76^i^ ± 0.06
GFRF72	5.50^c^ ± 0.00	1.13^def^ ± 0.06
UBRF	5.85^g^ ± 0.07	0.99^cde^ ± 0.13
GFBRF24	5.70^ef^ ± 0.00	0.90^cd^ ± 0.25
GFBRF48	5.65^de^ ± 0.07	1.17^ef^ ± 0.13
GFBRF72	5.45^bc^ ± 0.07	2.40^k^ ± 0.00

*Note:* Values with different superscript letters in the same column are significantly different (*p* < 0.05).

Abbreviations: GFBRF24, germinated‐fermented bran flour for 24 h; GFBRF48, germinated‐fermented bran flour for 48 h; GFBRF72, germinated‐fermented bran flour for 72 h; GFPG24, germinated‐fermented dehulled grain for 24 h; GFPG48, germinated‐fermented dehulled grain for 48 h; GFPG72, germinated‐fermented dehulled grain for 72 h; GFRF24, germinated‐fermented rootless flour for 24 h; GFRF48, germinated‐fermented rootless flour for 48 h; GFRF72, germinated‐fermented rootless flour for 72 h; GFWG24, germinated‐fermented grain for 24 h; GFWG48, germinated‐fermented grain for 48 h; GFWG72, germinated‐fermented grain for 72 h; TTA, titratable acidity; UBRF, unfermented‐germinated bran flour for 0 h; UGPG, unfermented‐germinated dehulled grain for 0 h; UGRF, unfermented‐germinated rootless flour for 0 h; UGWG, unfermented‐germinated grain for 0 h; UWGF, unfermented‐germinated whole grain for 0 h; WGFF24, germinated‐fermented whole grain for 24 h; WGFF48, germinated‐fermented whole grain for 48 h; WGFF72, germinated‐fermented whole grain for 72 h.

Titratable acidity values ranged from 0.57% to 2.40%, with sample GFBRF72 exhibiting the highest value of 2.40%. All the samples are significantly different from each other at *p* ≤ 0.05. The results showed that fermentation has a significant effect on the TTA values of the samples. TTA is a measure of the amount of acid present in a solution. The TTA of all germinated grain samples increased with an increase in fermentation time. The decrease in pH and the increase in TTA might be due to the degradation of some complex organic molecules, such as lipids, phytin, and proteins, into simpler compounds. The results obtained in this study agree with those of Owheruo et al. (2019), who reported a reduction in pH and an increase in TTA as malting progressed in finger and pearl millet.

### 3.3 Functional Properties of Germinated and Fermented Pigeon Pea Flour

The functional properties of germinated and fermented flours from pigeon pea are presented in Table 3. There were significant differences (*p* < 0.05) in the bulk densities of all the fermented and germinated pigeon pea flour samples (Table 3). There was a significant difference (*p* < 0.05) in the loose bulk density and packed bulk density of the samples, with values ranging from 0.392 to 0.593 and 0.540 to 0.744 g/cm^3^, respectively. Bulk density is dependent on the particle size of the sample. The reduction in bulk density, as observed in this study, agrees with the findings of Onimawo and Akubor (2005). This study reported a reduction in the bulk density of pigeon pea seed flour due to fermentation. Bulk density (BD) is utilized to assess flour heaviness, handling requirements, and the type of packaging materials suitable for the storage and transportation of food materials (Awuchi et al. 2019). A progressive increase in fermentation duration was observed to decrease the loose bulk density of all the samples. The tapped bulk density of germinated‐fermented dehulled grain (GFPG) and germinated‐fermented rootless flour (GFRF) samples was observed to decrease with an increase in fermentation duration. However, the 24‐h fermentation duration increased the tapped bulk density of the WGF, GFWG, and GFBRF samples, after which a decrease in the tapped bulk density values was observed with an increase in fermentation duration. A similar observation was reported by Ocheme et al. (2015) for the loose and packed bulk density of germinated sorghum flour. The reduction in bulk density (both loose and packed) observed may be due to the breakdown of complex compounds, such as starch and proteins, resulting from the modifications that occurred during germination (Ocheme et al. 2015). Reduced bulk density promotes easy digestibility of food products, especially in children with immature digestive systems. The reduced bulk density values in this study resulted in flour samples being packaged in smaller quantities with a constant volume, thereby reducing the costs associated with packaging.

**TABLE 3:** Function properties of germinated‐fermented pigeon pea flour samples.

Samples	LBD (g/cm^3^)	TBD (g/cm^3^)	WAC (g/g)	OAC (g/g)	SP (g/g)	SI (g/g)	FC (%)	DISP (%)
UWGF	0.43^c^ ± 0.01	0.54^a^ ± 0.02	3.06^e^ ± 0.01	1.28^fg^ ± 0.00	3.73^k^ ± 0.02	0.10^cd^ ± 0.01	4.00^a^ ± 0.00	63.00^i^ ± 1.41
WGFF24	0.41^b^ ± 0.00	0.62^b^ ± 0.01	3.01^e^ ± 0.00	1.21^ef^ ± 0.01	3.04^i^ ± 0.04	0.11^cd^ ± 0.02	14.00^ef^ ± 0.00	63.00^i^ ± 1.41
WGFF48	0.40^a^ ± 0.01	0.56^a^ ± 0.02	3.61^f^ ± 0.07	1.42^g^ ± 0.00	3.31^j^ ± 0.01	0.16^ef^ ± 0.01	12.00^cde^ ± 0.00	66.80^j^ ± 1.69
WGFF72	0.39^a^ ± 0.01	0.54^a^ ± 0.00	3.73^f^ ± 0.05	1.43^g^ ± 0.20	3.97^l^ ± 0.00	0.18^f^ ± 0.04	10.00^c^ ± 0.00	71.00^k^ ± 1.41
UGWG	0.56^k^ ± 0.00	0.67^cd^ ± 0.02	1.67^b^ ± 0.04	0.81^ab^ ± 0.07	2.43^g^ ± 0.04	0.05^ab^ ± 0.01	11.00^cd^ ± 1.41	41.00^bc^ ± 1.41
GFWG24	0.55^k^ ± 0.00	0.74^f^ ± 0.00	1.75^bc^ ± 0.05	0.73^a^ ± 0.03	2.10^d^ ± 0.03	0.06^ab^ ± 0.01	11.50^cd^ ± 0.70	35.00^a^ ± 1.41
GFWG48	0.52^ij^ ± 0.00	0.72^ef^ ± 0.01	1.88^cd^ ± 0.80	0.92^abcd^ ± 0.00	2.03^c^ ± 0.03	0.06^ab^ ± 0.02	12.00^cde^ ± 0.00	44.00^de^ ± 0.00
GFWG72	0.52^i^ ± 0.01	0.64^bc^ ± 0.00	1.92^d^ ± 0.05	1.06^de^ ± 0.08	1.97^b^ ± 0.01	0.10^cd^ ± 0.01	19.00^h^ ± 1.41	46.00^ef^ ± 0.00
UGPG	0.58^l^ ± 0.01	0.73^ef^ ± 0.02	1.89^cd^ ± 0.10	0.85^abcd^ ± 0.04	2.65^h^ ± 0.02	0.03^a^ ± 0.00	15.50^f^ ± 0.70	52.40^h^ ± 0.56
GFPG24	0.50^ghi^ ± 0.01	0.64^bc^ ± 0.05	1.88^cd^ ± 0.00	0.93^abcd^ ± 0.12	2.12^d^ ± 0.03	0.05^ab^ ± 0.01	16.00^fg^ ± 0.00	47.20^fg^ ± 1.13
GFPG48	0.50^fgh^ ± 0.00	0.63^bc^ ± 0.00	1.94^d^ ± 0.01	1.05^cde^ ± 0.06	2.40^fg^ ± 0.01	0.10^cd^ ± 0.03	13.00^de^ ± 1.41	45.00^def^ ± 1.41
GFPG72	0.49^ef^ ± 0.00	0.61^b^ ± 0.01	1.97^d^ ± 0.06	1.22^ef^ ± 0.33	2.62^h^ ± 0.03	0.13^de^ ± 0.04	6.50^b^ ± 0.70	44.00^de^ ± 0.00
UGRF	0.59^l^ ± 0.00	0.74^ef^ ± 0.01	1.53^a^ ± 0.00	0.84^abc^ ± 0.05	2.38^f^ ± 0.04	0.05^ab^ ± 0.02	12.00^cde^ ± 0.00	51.60^h^ ± 0.56
GFRF24	0.53^j^ ± 0.00	0.70^de^ ± 0.01	1.84^cd^ ± 0.04	0.92^abcd^ ± 0.09	2.22^e^ ± 0.02	0.06^ab^ ± 0.02	14.00^ef^ ± 0.00	48.40^g^ ± 0.56
GFRF48	0.49^fgh^ ± 0.00	0.67^cd^ ± 0.00	1.92^d^ ± 0.09	0.94^abcd^ ± 0.05	2.17^e^ ± 0.03	0.07^abc^ ± 0.01	18.00^gh^ ± 0.00	44.00^de^ ± 0.00
GFRF72	0.49^efg^ ± 0.01	0.65^bc^ ± 0.00	1.96^d^ ± 0.00	0.95^bcd^ ± 0.03	2.04^c^ ± 0.01	0.11^cd^ ± 0.01	20.00^h^ ± 0.00	43.60^de^ ± 0.56
UBRF	0.51^hi^ ± 0.01	0.61^b^ ± 0.00	1.46^a^ ± 0.15	0.78^ab^ ± 0.00	1.84^a^ ± 0.01	0.05^ab^ ± 0.01	13.00^de^ ± 1.41	39.60^b^ ± 0.56
GFBRF24	0.48^e^ ± 0.00	0.70^de^ ± 0.02	1.85^cd^ ± 0.03	0.79^ab^ ± 0.00	2.02^c^ ± 0.00	0.05^ab^ ± 0.01	18.00^gh^ ± 0.00	40.00^b^ ± 0.00
GFBRF48	0.45^d^ ± 0.01	0.67^cd^ ± 0.01	1.86^cd^ ± 0.02	0.91^abcd^ ± 0.01	2.05^c^ ± 0.01	0.05^ab^ ± 0.00	18.00^gh^ ± 0.00	43.00^cd^ ± 1.41
GFBRF72	0.45^d^ ± 0.01	0.65^bc^ ± 0.05	1.92^d^ ± 0.05	0.94^abcd^ ± 0.00	2.10^d^ ± 0.02	0.09^bcd^ ± 0.02	23.50^i^ ± 0.70	47.00^fg^ ± 1.41

*Note:* Values with different superscript letters in the same column are significantly different (*p* < 0.05).

Abbreviations: DISP, dispersibility; FC, foaming capacity; GFBRF24, germinated‐fermented bran flour for 24 h; GFBRF48, germinated‐fermented bran flour for 48 h; GFBRF72, germinated‐fermented bran flour for 72 h; GFPG24, germinated‐fermented dehulled grain for 24 h; GFPG48, germinated‐fermented dehulled grain for 48 h; GFPG72, germinated‐fermented dehulled grain for 72 h; GFRF24, germinated‐fermented rootless flour for 24 h; GFRF48, germinated‐fermented rootless flour for 48 h; GFRF72, germinated‐fermented rootless flour for 72 h; GFWG24, germinated‐fermented grain for 24 h; GFWG48, germinated‐fermented grain for 48 h; GFWG72, germinated‐fermented grain for 72 h; LBD, loose bulk density; OAC, oil absorption capacity; SI, solubility index; SP, swelling power; TBD, tapped bulk density; UBRF, unfermented‐germinated bran flour for 0 h; UGPG, unfermented‐germinated dehulled grain for 0 h; UGRF, unfermented‐germinated rootless flour for 0 h; UGWG, unfermented‐germinated grain for 0 h; UWGF, unfermented‐germinated whole grain for 0 h; WAC, water absorption capacity; WGFF24, germinated‐fermented whole grain for 24 h; WGFF48, germinated‐fermented whole grain for 48 h; WGFF72, germinated‐fermented whole grain for 72 h.

Water absorption capacity refers to the ability of flour to absorb water and swell, thereby improving the consistency of food (Awuchi et al. 2019). The results show an increase in water absorption as fermentation time increased, which is consistent with the findings of Adebowale and Maliki (2011) and Falmata et al. (2014). Both studies reported an increase in the WAC of sprouted, fermented, and combined sprouted and fermented sorghum, cowpea, and groundnut seeds. Additionally, it has been reported that boiling increases the water absorption capacities of legumes (Sobowale et al. 2023; Arukwe et al. 2017). There were significant differences (*p* < 0.05) in the water absorption capacities of all the samples of pigeon pea flour studied. In contrast, the values of ungerminated grains were higher than those of germinated grains (Table 3). A similar trend was observed by Pal et al. (2016) for germinated horse‐gram flour. Germinated samples at the same time of fermentation (24, 48, and 72 h) were not significantly different (*p* > 0.05). It was further observed that germinated samples at 48 and 72 h were not significantly different (*p* > 0.05). The increase in WAC can be attributed to the breakdown of polysaccharides into monosaccharides during germination, which increases the active sites for interaction between water and molecules, thereby contributing to the increase in WAC values (Handa et al. 2017). WAC describes flour's ability to associate with water under limited water supply, and this result suggests that fermented pigeon pea flour may find application in the production of certain baked products.

The OAC of the whole grain and germinated grain samples gradually increased with an increase in fermentation duration, as shown in Table 3. There were significant differences (*p* ≤ 0.05) in the oil absorption capacities among the germinated‐fermented samples of the pigeon pea flour. The OAC decreased with germination, as WGF samples had higher values compared to the malted samples. Samples at 24, 48, and 72 h (except for germinated‐fermented dehulled grain [GFPG]) were not significantly different (*p* > 0.05) from one another. OAC is the ability of flour samples to absorb oil (Awuchi et al. 2019). The result, however, does not correspond with that reported by Adebowale and Maliki (2011), who stated that fermentation decreases the OAC of pigeon pea flour. However, the result aligns with the report by Falmata et al. (2014), who found an increase in the OAC of cereal and legume seeds due to sprouting, fermentation, or a combination of both. Deepali et al. (2013) stated that the increase in OAC may be due to the solubilization and dissociation of proteins, leading to the exposure of nonpolar constituents from within the protein molecule. Oil binding enhances flavor and mouthfeel. Furthermore, foods with good oil‐binding abilities can be used as meat substitutes and extenders.

The results of this show that germination and fermentation generally decreased the swelling power of the flour samples as shown in Table 3. The swelling power ability of bran samples increased with increasing fermentation duration. The highest value of swelling capacity (3.97 g/g) was recorded for whole grain germinated‐fermented flour for 72 h (WGFF72). There were significant differences (*p* ≤ 0.05) between the swelling capacity of fermented and germinated pigeon samples. Swelling power, which is the measure of the starch's ability in flour or food to absorb water and swell (Olatunde et al. 2017). The increase in swelling capacity of pigeon pea flour processed with these methods may be due to modifications to the starch granules, resulting in higher water uptake by the granules. This result aligns with the reports by Falmata et al. (2014), who stated that sprouting, fermentation, and the combination of sprouting and fermentation of legumes and cereals enhance their swelling capacities. For whole grain and peeled grain, the 24‐h fermentation duration decreased the swelling power ability of the samples, after which the values increased gradually with an increase in germination duration. However, swelling power for fermented‐germinated grains and rootless grains decreased with an increase in fermentation duration. Variations observed in the swelling power of different grain samples are consistent with those reported by various authors. Ocheme et al. (2015) reported that the swelling power of germinated finger millet flour increased with the duration of germination. They attributed this increase in soluble solids to the breakdown of lipids, fiber, and a larger amount of amylose‐lipid complex in flour, which could inhibit the swelling of starch granules during germination. On the other hand, Adedeji et al. (2014) reported that the swelling power of germinated maize flour increased with an increase in germination time, which they attributed to the disruption of hydrogen bonds in the flour samples by amylases and proteases being hydrolyzed into sugars and amino acids. A low swelling capacity is beneficial for handling food in the gut, especially in infants (Ocheme et al. 2015). The solubility index is the property of solid, liquid, or gaseous food (chemical) substances known as solutes that dissolve in a liquid, gaseous, or solid solvent (Awuchi et al. 2019).

The solubility index of all the grain samples increased with a progressive increase in germination duration. There was a significant difference (*p* < 0.05) among the samples. The increase in solubility index can be attributed to the breakdown of polysaccharides into monosaccharides during germination (Handa et al. 2017). A similar result was obtained by Handa et al. (2017) for germinated horse‐gram flour. There were significant differences (*p* ≤ 0.05) in the foam capacities of the germinated‐fermented pigeon pea flour samples. The foaming capacity refers to the amount of interfacial area that the protein can create. They are functions of a type of protein, pH, processing methods, viscosity, and surface tension (Fekria et al. 2012). A significant difference (*p* < 0.05) exists among the samples. The bran sample fermented for 72 h had the highest value (23.50%), while the lowest value (4.00%) was observed in the unfermented whole grain. The foaming capacity of germinated‐fermented grain, rootless grain, and bran samples was observed to increase with increased fermentation duration. However, it was observed that the fermentation duration of 24 h increased the foaming capacity of whole grain and peeled germinated samples, after which the values decreased with an increase in germination duration. The trends recorded in this study align with the report by Adedeji et al. (2014), who found that the foaming capacity of germinated maize flour decreased with increasing germination time. Food materials with good foaming capacity are helpful in the formulation of aerated foods (Ocheme et al. 2015). In addition, the foam capacity values obtained in this study were close to those reported for pumpkin (13.2%) and germinated tiger nut varieties (4.00%–11.33%) by Chinma et al. (2009). Foam formation and stability are functions of the type of protein, pH, processing methods, viscosity, and surface tension (Arukwe et al. 2017).

Dispersibility is a measure of the reconstitution of flour in water (Otegbayo et al. 2013). There was a significant difference (*p* < 0.05) among the samples. The highest value was observed in whole grain germinated and fermented for 72 h, while the lowest value was observed in germinated and fermented grain for 24 h. The dispersibility of whole grain, germinated grain, and bran samples increased with an increase in fermentation duration. At the same time, the values were observed to decrease with an increase in fermentation duration for peeled germinated and rootless grains. A trend was reported by Pal et al. (2016) for the increasing use of germinated horse‐gram flour.

### 3.4 Color Properties of Germinated and Fermented Pigeon Pea Flour

The changes in color of fermented and germinated pigeon pea flour are presented in Table 4 and were expressed as L*, a*, and b* values. Color is one of the most important attributes of food materials, as it influences consumer acceptability; hence, color serves as a process quality control (Pankaj et al. 2013). The lightness index (L*) of fermented and germinated pigeon pea flour ranged from 51.30 to 79.81, redness (a*) ranged between −0.86 and −7.22, yellowness (b*) ranged from 13.91 to 20.43, change in lightness (*∆L*) ranged from 38.07 to 66.57, change in redness (*∆a*) ranged from −1.34 to −7.70, change in yellowness (*∆b*) ranged from 11.55 to 19.33, while the total color difference (*∆E*) ranged between 39.84 and 69.58. The color attributed to fermented and germinated pigeon pea flour showed a significant difference (*p* < 0.05) as influenced by duration. The results show that as the fermentation period increases, the lightness (L*) of the whole grain germinated‐fermented flour decreases (WGFF) from 75.55 to 51.46, which is similar to Chinma et al. (2021), who reported that fermentation significantly decreased the L* value of Bambara groundnut flour samples. Fermentation duration increased the lightness of germinated‐fermented whole pigeon pea flour (GFWG) from 59.23 to 75.49. However, the fermentation–germination duration increased the lightness of germinated, peeled, and rootless flour but reduced the lightness of the bran flour. The negative value observed for redness (a*) in this study indicates that the flours were greenish. A reduction was observed for fermented whole pigeon pea flour, while an increased value was recorded for all the fermented‐germinated flours. Jan et al. (2016) recorded a decrease in L* value from 59.2 to 6.2 with a corresponding increase in a* from 0.1 to 0.6 and yellowness (b*) from 9.3 to 11.8 value after germination of *Chenopodium* seed flour. The “L*” is regarded as the measure of the degree of redness or greenness, while the “b*” represents the degree of yellowness or blueness in a material. The “a*” is negative, thus indicating the absence of red or green color. The findings further support the b* value recorded in this study because it was observed that fermentation increased the yellowness of whole pigeon pea from 14.13 to 19.62 and fermented‐germinated rootless flour from 18.63 to 20.43. Tian et al. (2010) stated that the reduction in L* and subsequent increase in a* and b* values in germinated flour were attributed to the formation of starch and protein, together with the Maillard reaction between the two constituents during the drying of flour. This could be the case for an increase observed in the b* value of germinated whole pigeon pea flour in this study.

**TABLE 4:** Color properties of germinated‐fermented pigeon pea flour samples.

Material	L*	a*	b*	*∆L*	*∆a*	*∆b*	*∆E*
UWGF	75.55^b^ ± 1.75	−6.91^kl^ ± 0.17	19.34^cd^ ± 0.49	62.32^b^ ± 1.74	−7.38^kl^ ± 0.17	18.23^c^ ± 0.49	65.35^bc^ ± 1.82
WGFF24	70.61^d^ ± 0.52	−5.87^g^ ± 0.04	17.31^hi^ ± 0.14	57.37^d^ ± 0.53	−6.35^g^ ± 0.04	16.20^hi^ ± 0.14	59.95^f^ ± 0.54
WGFF48	51.30^i^ ± 0.44	−1.75^b^ ± 0.01	12.66^k^ ± 0.11	38.07^i^ ± 0.43	−2.23^b^ ± 0.01	11.55^k^ ± 0.11	39.84^k^ ± 0.45
WGFF72	51.46^i^ ± 0.06	−0.86^a^ ± 0.02	12.96^k^ ± 0.02	38.22^i^ ± 0.06	−1.34^a^ ± 0.02	11.85^k^ ± 0.02	40.05^k^ ± 0.07
UGWG	59.32^g^ ± 0.27	−2.71^c^ ± 0.06	14.13^j^ ± 0.04	46.09^g^ ± 0.27	−3.19^c^ ± 0.06	13.02^j^ ± 0.04	48.00^i^ ± 0.27
GFWG24	70.77^d^ ± 0.65	−6.58^ij^ ± 0.03	18.58 ± 0.16	57.53^d^ ± 0.65	−7.06^ij^ ± 0.04	17.48^f^ ± 0.17	60.55^ef^ ± 0.66
GFWG48	75.61^b^ ± 0.58	−7.02^l^ ± 0.34	19.62^bc^ ± 0.16	62.37^b^ ± 0.58	−7.50^l^ ± 0.34	18.52^bc^ ± 0.17	65.49^bc^ ± 0.56
GFWG72	75.49^b^ ± 1.72	−6.52^hi^ ± 0.17	18.18^g^ ± 0.40	62.26^b^ ± 1.73	−7.01^hi^ ± 0.17	17.10^g^ ± 0.38	64.94^c^ ± 1.77
UGPG	76.19^b^ ± 0.31	−6.39^hi^ ± 0.03	19.95^b^ ± 0.07	62.96^b^ ± 0.31	−6.87^hi^ ± 0.03	18.85^b^ ± 0.07	66.07^bc^ ± 0.32
GFPG24	66.09^f^ ± 1.31	−5.13^de^ ± 0.07	17.61^h^ ± 0.35	52.86^f^ ± 1.30	−5.61^de^ ± 0.07	16.50^h^ ± 0.34	55.66^h^ ± 1.34
GFPG48	79.81^a^ ± 0.52	−7.02^l^ ± 0.04	19.89^b^ ± 0.13	66.57^a^ ± 0.52	−7.49^l^ ± 0.04	18.79^b^ ± 0.13	69.58^a^ ± 0.53
GFPG72	71.93^d^ ± 1.26	−6.35^h^ ± 0.09	18.61^f^ ± 0.34	58.70^d^ ± 1.25	−6.83^h^ ± 0.09	17.50^f^ ± 0.34	61.63^e^ ± 1.29
UGRF	76.01^b^ ± 0.11	−6.56^ij^ ± 0.02	18.63^f^ ± 0.04	62.77^b^ ± 0.11	−7.05^ij^ ± 0.03	17.53^f^ ± 0.04	65.55^bc^ ± 0.11
GFRF24	66.75^ef^ ± 0.66	−5.00^d^ ± 0.04	17.16^i^ ± 0.18	53.51^ef^ ± 0.66	−5.48^d^ ± 0.05	16.39^h^ ± 0.43	56.14^h^ ± 0.68
GFRF48	73.29^c^ ± 0.75	−6.74^jk^ ± 0.22	19.06^de^ ± 0.15	60.06^c^ ± 0.75	−7.22^jk^ ± 0.22	17.95^de^ ± 0.15	63.10^d^ ± 0.78
GFRF72	76.53^b^ ± 0.19	−7.22^m^ ± 0.01	20.43^a^ ± 0.05	63.29^b^ ± 0.19	−7.70^m^ ± 0.01	19.33^a^ ± 0.05	66.63^b^ ± 0.19
UBRF	67.45^ef^ ± 0.08	−5.46^f^ ± 0.04	17.01^i^ ± 0.05	54.21^ef^ ± 0.08	−5.94^f^ ± 0.04	15.91^i^ ± 0.05	56.81^gh^ ± 0.08
GFBRF24	67.81^eb^ ± 0.45	−5.27^ef^ ± 0.03	19.26^d^ ± 0.21	54.57^e^ ± 0.45	−5.73^e^ ± 0.03	18.16^cd^ ± 0.20	57.80^g^ ± 0.49
GFBRF48	70.60^d^ ± 0.04	−5.00^d^ ± 0.01	18.75^ef^ ± 0.01	57.36^d^ ± 0.04	−5.48^d^ ± 0.00	17.64^ef^ ± 0.01	60.26^ef^ ± 0.05
GFBRF72	55.57^h^ ± 0.01	−1.61^b^ ± 0.08	13.91^j^ ± 0.01	42.34^h^ ± 0.01	−2.08^b^ ± 0.08	12.81^j^ ± 0.01	44.27^j^ ± 0.01

*Note:* Values with different superscript letters in the same column are significantly different (*p* < 0.05).

Abbreviations: a*, redness; b*, yellowness; GFBRF24, germinated‐fermented bran flour for 24 h; GFBRF48, germinated‐fermented bran flour for 48 h; GFBRF72, germinated‐fermented bran flour for 72 h; GFPG24, germinated‐fermented dehulled grain for 24 h; GFPG48, germinated‐fermented dehulled grain for 48 h; GFPG72, germinated‐fermented dehulled grain for 72 h; GFRF24, germinated‐fermented rootless flour for 24 h; GFRF48, germinated‐fermented rootless flour for 48 h; GFRF72, germinated‐fermented rootless flour for 72 h; GFWG24, germinated‐fermented grain for 24 h; GFWG48, germinated‐fermented grain for 48 h; GFWG72, germinated‐fermented grain for 72 h; L*, lightness; UBRF, unfermented‐germinated bran flour for 0 h; UGPG, unfermented‐germinated dehulled grain for 0 h; UGRF, unfermented‐germinated rootless flour for 0 h; UGWG, unfermented‐germinated grain for 0 h; UWGF, unfermented‐germinated whole grain for 0 h; WGFF24, germinated‐fermented whole grain for 24 h; WGFF48, germinated‐fermented whole grain for 48 h; WGFF72, germinated‐fermented whole grain for 72 h.

### 3.5 Antinutrient Composition of Germinated and Fermented Pigeon Pea Flour

The antinutrient content of both fermented and germinated pigeon pea flour is represented in Table 5. Antinutritional factors in foods are primarily responsible for negatively impacting the absorption of nutrients and micronutrients in the digestive system, which may interfere with the functioning of specific organs (Gemede and Ratta 2014). Ohanenye et al. (2022) suggested that tannins form insoluble complexes with proteins, which reduce protein digestibility by inactivating digestive enzymes and interacting with ionizable iron. The results show that the tannin value of whole grain germinated‐fermented flour (WGFF), germinated‐fermented whole grain flour (GFWG), germinated‐fermented dehulled grain (GFPG), germinated‐fermented rootless flour (GFRF), and germinated‐fermented bran flour (GFBRF) fermented for 0, 24, 48, and 72 h ranged from 2.02 to 17.94 mg/100 g, 2.58 to 10.34 mg/100 g, 6.46 to 10.16 mg/100 g, 8.31 to 11.26 mg/100 g, and 1.38 to 19.64 mg/100 g, respectively. The results revealed that the tannin content was significantly lower (2.03 mg/100 g) in the 72‐h fermentation of the WGFF sample compared to the values at 0, 24, and 48 h. The GFPG tannin results show that unfermented‐germinated dehulled grain (UGPG) had the highest tannin value (10.16 mg/100 g). The GFPG 24 had the lowest tannin value (6.46 mg/100 g) compared to the other samples. Unfermented bran flour (UBRF) decreases from 19.64 mg/100 g to 1.38 mg/100 g after 24 h. GFRF and GFRF72 had the lowest value at 8.31 mg/100 g. The study exhibits a similar trend to that observed by Chinma et al. (2021), which reported a reduction in tannin content from 5.13 to 1.86 mg/100 g across various germination durations. Tannins present in food have a negative impact on protein digestibility in both animals and humans, rendering proteins partially inaccessible or inhibiting the action of digestive enzymes (Yao et al. 2019). The fermented and germinated pigeon pea flours in this study showed a low tannin content and were significantly different (*p* < 0.05). According to Akubor (2017), tannins have also been reported to reduce the digestibility and palatability of proteins and carbohydrates by forming insoluble complexes, thereby diminishing mineral bioavailability.

**TABLE 5:** Antinutrient composition of germinated pigeon pea flour samples.

Samples	Tannin (mg/100 g)	Phytate (mg/100 g)	Oxalate (mg/100 g)
UWGF	16.16^i^ ± 0.80	1.13^c^ ± 0.02	3.24^i^ ± 0.11
WGFF24	17.94^j^ ± 0.66	0.97^a^ ± 0.03	2.67^h^ ± 0.07
WGFF48	7.94^cde^ ± 0.63	1.01^ab^ ± 0.03	1.29^b^ ± 0.01
WGFF72	2.03^a^ ± 0.63	1.04^ab^ ± 0.02	0.85^a^ ± 0.03
UGWG	2.58^a^ ± 0.32	1.46^e^ ± 0.07	1.20^b^ ± 0.16
GFWG24	10.34^gh^ ± 0.84	1.10^b^ ± 0.05	3.57^j^ ± 0.10
GFWG48	4.61^b^ ± 0.84	1.05^ab^ ± 0.00	2.71^h^ ± 0.06
GFWG72	9.23^egh^ ± 1.60	1.25^cd^ ± 0.03	2.39^f^ ± 0.66
UGPG	10.16^fgh^ ± 0.84	1.45^e^ ± 0.05	2.60^h^ ± 0.07
GFPG24	6.46^c^ ± 0.84	1.11^b^ ± 0.02	1.62^c^ ± 0.04
GFPG48	9.42^efg^ ± 0.55	1.50^ab^ ± 0.11	2.44^fg^ ± 0.03
GFPG72	9.60^efg^ ± 1.39	1.14^b^ ± 0.03	1.84^d^ ± 0.14
UGRF	8.31^de^ ± 0.00	1.81^ij^ ± 0.03	3.34^i^ ± 0.07
GFRF24	11.26^h^ ± 0.31	1.27^d^ ± 0.11	1.62^c^ ± 0.12
GFRF48	8.68^def^ ± 0.63	1.60^fgh^ ± 0.09	1.61^c^ ± 0.02
GFRF72	8.31^de^ ± 1.10	1.31^d^ ± 0.13	2.56^g^ ± 0.10
UBRF	19.64^k^ ± 0.43	1.91^j^ ± 0.07	2.01^e^ ± 0.04
GFBRF24	1.38^cd^ ± 1.59	1.70^hi^ ± 0.13	3.90^k^ ± 0.09
GFBRF48	8.68^def^ ± 0.63	1.54^efg^ ± 0.07	2.36^f^ ± 0.01
GFBRF72	8.68^def^ ± 0.63	1.66^gh^ ± 0.06	0.91^a^ ± 0.08

*Note:* Values with different superscript letters in the same column are significantly different (*p* < 0.05).

Abbreviations: GFBRF24, germinated‐fermented bran flour for 24 h; GFBRF48, germinated‐fermented bran flour for 48 h; GFBRF72, germinated‐fermented bran flour for 72 h; GFPG24, germinated‐fermented dehulled grain for 24 h; GFPG48, germinated‐fermented dehulled grain for 48 h; GFPG72, germinated‐fermented dehulled grain for 72 h; GFRF24, germinated‐fermented rootless flour for 24 h; GFRF48, germinated‐fermented rootless flour for 48 h; GFRF72, germinated‐fermented rootless flour for 72 h; GFWG24, germinated‐fermented grain for 24 h; GFWG48, germinated‐fermented grain for 48 h; GFWG72, germinated‐fermented grain for 72 h; UBRF, unfermented‐germinated bran flour for 0 h; UGPG, unfermented‐germinated dehulled grain for 0 h; UGRF, unfermented‐germinated rootless flour for 0 h; UGWG, unfermented‐germinated grain for 0 h; UWGF, unfermented‐germinated whole grain for 0 h; WGFF24, germinated‐fermented whole grain for 24 h; WGFF48, germinated‐fermented whole grain for 48 h; WGFF72, germinated‐fermented whole grain for 72 h.

In this study, a significant difference (*p* < 0.05) in phytate content was observed among the samples. Whole grain flour (WGF) samples recorded 0.97 to 1.13 mg/100 g, which were significantly lower than those of germinated grain (GWG) samples. The phytate content decreased during the 24‐h fermentation period. For GWG samples, the phytate content decreased from 1.46 to 1.05 mg/100 g after 48 h of fermentation, before a slight increase to 1.25 mg/100 g in 72 h. The trend suggests that a more extended fermentation period results in lower phytate levels. Nwosu et al. (2013) reported a similar range (0.61–1.10 mg/100 g) for germinated pigeon pea. The phytate reported in this study was lower compared to the study of Feizollahi et al. (2021), who reported phytate values for Bambara groundnut to be (1.35–4.81 mg/100 g). Phytate, also known as inositol hexakisphosphate (IP6), is a potent antinutritional factor in plant feeds, present in significant quantities in major legumes and oilseeds (Gemede and Ratta 2014). Phytic acid exhibits an inhibitory effect on gastrointestinal enzymes, including tyrosinase, trypsin, pepsin, lipase, and amylase (Feizollahi et al. 2021). In germinated‐fermented dehulled grain (GFPG) samples, the phytate content decreased from 1.45 mg/100 g to 1.11 mg/100 g during a 24‐h fermentation period. Similar trends occurred in germinated‐fermented rootless flour (GFRF) and germinated‐fermented bran flour (GFBRF) samples. These values ranged from 1.27 to 1.60 mg/100 g for GFRF samples and from 1.70 to 1.66 mg/100 g for GFBRF flour during the fermentation periods. Nwosu et al. (2013) noted that the reduction in phytate content in germination samples compared with the raw sample could be due to the time of sprouting and germination, as well as the seed coat, which is dehulled after drying. Feizollahi et al. (2021) stated that the most significant effect of phytic acid on human nutrition is its reduction of zinc bioavailability. Phytate content in food can be lowered by the addition of enzymes (phytase) that hydrolyze it.

The oxalate content of pigeon pea flours exhibited significant differences (*p* < 0.05) in both fermented and germinated samples, ranging from 0.85 to 3.24 mg/100 g in WGF samples. Oxalate contents in the WGFF samples decreased with increasing fermentation duration (0–72 h). Conversely, the oxalate content in GFWG flour increased from 1.20 to 3.57 mg/100 g within the first 24 h of germination, with a subsequent decrease between 48 and 72 h. The oxalate values in this study were notably higher than the 0.64–1.01 mg/100 g reported for germinated pigeon pea flour at 24 and 48 h (Nwosu et al. 2014). Germination was observed to influence the oxalate content of GPG, GRF, and BRF samples in this study. The lowest oxalate content was found in BRF after 72 h. Oxalates in food can form complexes with dietary minerals, making them less available to humans and reducing their bioavailability. Germination has been suggested to reduce oxalate, thereby enhancing the nutritional value of the seeds (Nwosu et al. 2014).

## 4 Conclusion

The study demonstrated the effect of germination and the subsequent fermentation period on pigeon pea and its structural parts. The findings show that germination and fermentation enhanced the nutritional content of the pigeon pea flour, especially the protein content, with germinated‐fermented bran flour (GFBRT72) having the highest protein content. Additionally, germinated and fermented pigeon pea flour significantly reduced the antinutrient levels. Also, the color properties of pigeon pea flour were positively influenced by treatment type and duration; the treatment and duration positively influenced the functional properties of pigeon pea samples. Overall, this research highlights the promising potential of germinated and fermented pigeon pea flour and its structural parts as a sustainable, nutrient‐rich food source in addressing protein‐energy malnutrition, and its versatility in developing infant and baked food products.

## Author Contributions

**Sunday Samuel Sobowale:** conceptualization (equal), funding acquisition (equal), investigation (equal), methodology (equal), project administration (equal), resources (equal), software (equal), supervision (equal), validation (equal), writing – original draft (equal), writing – review and editing (equal). **Bukola Funmilola Olatunji:** data curation (equal), formal analysis (equal), investigation (equal), methodology (equal), validation (equal), visualization (equal), writing – original draft (equal). **Olanrewaju Emmanuel Fayemi:** project administration (equal), resources (equal), validation (equal), visualization (equal), writing – review and editing (equal).

## Conflicts of Interest

The authors declare no conflicts of interest.

## Data Availability

The data that support the findings of this study are available from the corresponding author upon reasonable request.
